# Cultured Human Airway Epithelial Cells (Calu-3): A Model of Human Respiratory Function, Structure, and Inflammatory Responses

**DOI:** 10.1155/2010/394578

**Published:** 2010-06-27

**Authors:** Yan Zhu, Aaron Chidekel, Thomas H. Shaffer

**Affiliations:** ^1^Nemours Biomedical Research, Nemours Research Lung Center, Alfred I. duPont Hospital for Children, 1600 Rockland Road, Wilmington, DE 19803, USA; ^2^Department of Pediatrics, Jefferson Medical College, Thomas Jefferson University, 1025 Walnut Street, Suite 700, Philadelphia, PA 19107, USA; ^3^Department of Pediatrics, Alfred I. duPont Hospital for Children, 1600 Rockland Road, Wilmington, DE 19803, USA; ^4^Departments of Physiology and Pediatrics, School of Medicine, Temple University, 3420 North Broad Street, Philadelphia, PA 19140, USA

## Abstract

This article reviews the application of the human airway Calu-3 cell line as a respiratory model for studying the effects of gas concentrations, exposure time, biophysical stress, and biological agents on human airway epithelial cells. Calu-3 cells are grown to confluence at an air-liquid interface on permeable supports. To model human respiratory conditions and treatment modalities, monolayers are placed in an environmental chamber, and exposed to specific levels of oxygen or other therapeutic modalities such as positive pressure and medications to assess the effect of interventions on inflammatory mediators, immunologic proteins, and antibacterial outcomes. Monolayer integrity and permeability and cell histology and viability also measure cellular response to therapeutic interventions. Calu-3 cells exposed to graded oxygen concentrations demonstrate cell dysfunction and inflammation in a dose-dependent manner. Modeling positive airway pressure reveals that pressure may exert a greater injurious effect and cytokine response than oxygen. In experiments with pharmacological agents, Lucinactant is protective of Calu-3 cells compared with Beractant and control, and perfluorocarbons also protect against hyperoxia-induced airway epithelial cell injury. The Calu-3 cell preparation is a sensitive and efficient preclinical model to study human respiratory processes and diseases related to oxygen- and ventilator-induced lung injury.

## 1. Introduction

The airway epithelial is involved in the pathogenesis and treatment of many lung diseases, including adult and neonatal respiratory distress syndrome (RDS), chronic obstructive pulmonary disease (COPD), asthma, the adverse effects of positive pressure mechanical ventilation [1–4], and supplemental oxygen therapy [5]. The airway epithelium is directly exposed to the external environment and is highly responsive to biophysical and biological stimuli. Via a variety of cellular pathways and mechanisms, the respiratory epithelium responds to exogenous substances, stresses, or medical therapies that may promote airway repair or injury. 

Understanding the role of the airway epithelium and its response to injurious stimuli, therapeutic drugs, and interventions will aid in determining the mechanisms of airway injury and should facilitate the development of novel therapies for lung diseases. This experimental approach is one component of a robust approach to assess multiple aspects of respiratory pathophysiology. With the inherent difficulties of primary human lung cell culture and the limit of whole-animal studies, *in vitro* models have been developed to study the mechanism of hyperoxia or positive pressure-induced lung injury and to evaluate any new therapies [6, 7].

Although numerous *in vitro* models have been explored to study the airway epithelium response, we have focused on the Calu-3 cell line for the following reasons. (1) The Calu-3 cell line (American Type Culture Collection, ATCC HTB-55) is a well-differentiated and characterized cell line, that is derived from human bronchial submucosal glands [8]. In the human lung, the submucosal glands are a major source of airway surface liquid, mucins, and other immunologically active substances, and Calu-3 cells reflect these properties [9–12]. By contrast, other pulmonary cell types, such as airway columnar cells and type II (alveolar) pneumocytes, are relatively less active from a secretory standpoint and have less impact on the composition of airway surface liquid. (2) Calu-3 cells are grown at an air-liquid interface and demonstrate many characteristics of the bronchiolar epithelium, which, *in vivo,* serves as the barrier layer between inspired gas and other visceral tissues. This attribute is particularly advantageous for the evaluation of airway injury and response to medical treatments and respiratory therapeutic interventions. Air-liquid interface culture enables the approximation of the *in vivo* situation of mechanical ventilation and oxygen toxicity better than other *in vitro* models [13], such as those using lung explants or submerged primary cell culture systems. Calu-3 cells have been used for drug delivery [14], pulmonary drug disposition [7], and bacterial invasiveness studies [15]. Devor et al. have employed the Calu-3 cell line as a model system to study the mechanisms of HCO_3_^−^ and Cl^−^ secretion, which reflects the transport properties of native submucosal gland serous cells [16].

We have developed a sensitive and efficient model using Calu-3 cells cultured in an environmental chamber as a respiratory model to study the effects of oxygen concentration and exposure time [5], biophysical stresses, and biological agents [10, 12, 13], such as perfluorocarbons (PFC) and surfactant. PFC is known to exert numerous effects *in vitro* and *in vivo*. Partial liquid ventilation with PFC attenuates oxidative injury in experimental models of lung injury [17, 18]. PFC liquid also attenuates oxidative damage to both biologic (rat endothelial cells) and nonbiologic (linoleic acid in sodium dodecyl sulfate micelles) systems [19]. Surfactant-associated proteins play an important role in the maintenance of pulmonary homeostasis. The deficiency of surfactant-associated proteins is associated with severe respiratory distress, and, in the case of surfactant protein B (SP-B), is incompatible with life [20]. Sinulpeptide (KL-4), a synthetic peptide that functionally mimics SP-B [21], prevents hyperoxic and lipopolysaccharide-induced lung injury in mice [22]. An animal study demonstrated inflammation and lung function in adult mice with SP-B deficiency and reversal of inflammation and maintenance of lung function with SP-B administration *in vivo *[23].

Apical fluid and protein secretions in Calu-3 cultures are similar to those from primary airway cultures and model many aspects of the intraluminal condition in the conducting airways of the lung. This model allows for the analysis of airway tissue tolerance of oxygen exposure without the confounding variables of a whole-animal model. 

In these *in vitro* experiments, clinically relevant outcomes were measured to assess the effect of respiratory interventions. Since airway inflammation is commonly a result of barotrauma and oxygen toxicity, ELISA techniques detect changes in the proinflammatory cytokines IL-6 and IL-8. Pulmonary edema also results from airway injury, and, to model this outcome, total protein is measured in the apical wash fluid. Similarly, Calu-3 monolayer integrity and cellular physiology are assessed by measuring changes in transepithelial electrical resistance (TER). In previous studies, we found that TER correlates with FITC dextran flux from the basolateral to the apical aspect of the monolayer [24]. Finally, histological outcomes are assessed semiquantitatively by individual cell and monolayer morphology, as well as by quantifying cell viability.

This article introduces specific experimental methods for inducing environmental changes on Calu-3 cells, which are similar to clinical interventions practiced in respiratory management. In this regard, the methods discussed enable the study of changes in environment, gas concentrations, biophysical stresses, and therapeutic interventions on airway epithelial cells as a function of time and treatment.

## 2. Materials and Methods

### 2.1. Calu-3 Cell Culture

Calu-3 cell culture techniques are well described elsewhere [5, 12, 13]. The cells used for the experiments were 5–10 passages.

### 2.2. Collection of Calu-3 Apical Surface Fluid Washings

Apical surface fluid washings are the source of biologically active substances secreted apically by Calu-3 cells, and this fluid is used to model the airway compartment of the lung. A sample of the Calu-3 apical surface fluid (ASF) is collected from each transwell insert for each experimental condition so that the response of individual monolayers is assayed. Experiments are designed so that each monolayer is used only once. Therefore, it is necessary to use multiple plates over the course of each experiment. For example, if the response of Calu-3 monolayers to a specific concentration of oxygen is assayed over a 48-hour period, three plates are used. The first plate is used for the control condition, the second for 24-hour outcomes, and the third for outcomes at 48 hours.

ASF from Calu-3 cells is collected in the following fashion [5, 12]. The apical surface of the monolayer is gently washed twice with 140 *μ*L of sterile normal saline. Washes are combined for a total of 280 *μ*L for each individual monolayer per sample. Samples are centrifuged for 15 minutes at 13,000 rcf and at 4°C, and the supernatants stored in aliquots at −70°C for subsequent analysis. The levels of IL-6 and IL-8 in Calu-3 ASF were measured with quantitative ELISA using human IL-6 and IL-8 Quantikine ELISA kits (R & D Systems, Minneapolis, MN). Calu-3 ASF washings used in the assay were appropriately diluted. All standards and samples were assayed in duplicate. The test sensitivity for respective immunoassays was as follows: IL-6 (0.039 pg/mL) and IL-8 (10 pg/mL). Interassay and intraassay coefficients of variance are less than 10%.

### 2.3. Histology, Cell Viability, and Cytomorphology of Calu-3 Cells

**Figure 1:**
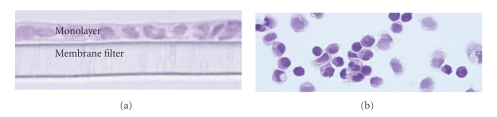
Representative normal morphology of Calu-3 cells. The sections of monolayer (a) and cytospins of Calu-3 cells (b) were stained with hematoxylin and eosin (*n* = 6 for each condition). All slides were examined by light microscopy at 40x magnification.

After exposing Calu-3 monolayers to an experimental condition or treatment, Calu-3 cell histology and viability are assessed. To evaluate Calu-3 monolayers in situ, transwell inserts containing Calu-3 monolayers are fixed in 4% paraformaldehyde. Following fixation, polycarbonate filters with the adherent monolayers are separated from the transwell inserts and embedded in paraffin. Cross-sections of the monolayer/filter samples are sectioned (5 *μ*m thick) and stained with hematoxylin and eosin (H&E) for histological analysis (Figure 1(a)).

Cell viability and cytomorphological studies are performed using single cell suspensions (SCS) obtained by harvesting the cells from transwell filters by trypsinization. Using 20 *μ*L of the SCS, cell viability is determined using the Trypan blue-exclusion assay. Cytospin slides are prepared using an additional 100 *μ*L of the SCS and fixed in formalin and stained with H&E (Figure 1(b)). Cytomorphology is evaluated in a semi-quantitative manner as the ratio of abnormal cells to total cells [5]. Abnormal cells are defined as cells demonstrating any of the following characteristics: (1) abnormal cellular appearance, (2) swollen nuclei, (3) intracellular and nuclear vacuoles, or (4) diffuse cytoplasm and cellular debris [5], or a combination of the four.

### 2.4. Set-Up of the Modular Incubator Chamber (MIC-101)

**Figure 2:**
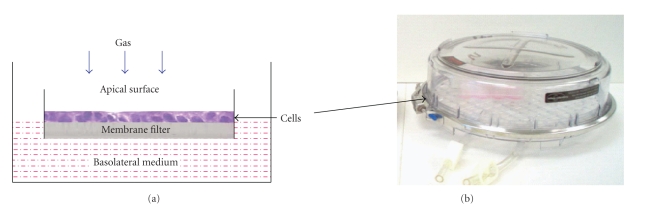
Calu-3 air-liquid interface cell culture in an environmental chamber. Calu-3 cell monolayers were grown at an air-liquid interface to full confluence before exposure to oxygen, positive pressure, and biological agents. Modular incubator chamber shown on the right; transwell inserts shown on the left.

To establish specific environmental conditions for the experiments described below, a sterile modular incubator chamber (MIC-101; Billups-Rothenberg, Del Mar, CA) is used. Cell culture plates are placed into the chamber, and it is sealed with Dow Corning high vacuum grease (Dow Corning, Midland, MI). With the chamber sealed and airtight, it is ready to be pressurized to a maximum of 2 pounds per square inch (PSI). To finalize the chamber setup, the appropriate devices and gas sources with or without a humidifier are attached to the chamber inlet. A three-way stopcock is used to attach a pressure gauge, oxygen analyzer, or flow meter to the outlet of the chamber (Figure 2).

### 2.5. Oxygen Concentration

Supplemental oxygen administration is an important component of patient care in respiratory medicine and is critical to the maintenance of organ homeostasis and neurological function; however, oxygen can be toxic to tissues of the lung and airway. The mechanisms of this toxicity include oxidative injury with the generation of free radicals and direct disruption of cell membranes. Strategies to control oxygen concentration and exposure time are employed clinically as a means to control airway oxidative injury. In the laboratory, it is possible to study the thresholds and exposure times that can be detrimental to airway epithelial cells, and these findings may provide clinically relevant insights into bedside management. 

The effect of oxygen concentration on Calu-3 cell structure and function is assessed in the following manner [5]. Calu-3 monolayers that exhibit TER values of at least 1,000 ohm·cm^2^ are exposed to normoxia (FiO_2_ = 21%, FiCO_2_ = 5%, balance nitrogen) or graded hyperoxia (FiO_2_ = 40%, 60%, 80%, FiCO_2_ = 5%, balance nitrogen) using the chamber described above. To obtain the desired oxygen concentration, the inlet and outlet ports of the chamber are opened and attached to a Servo O_2_-air 960 mixer (Siemens-Elema, Sweden), a 95% oxygen tank, 5% CO_2_, and air balance tank. The blended gas is warmed and humidified with an MR730 respiratory humidifier (Fisher & Paykel Healthcare, Laguna Hills, CA). The appropriate gas concentration is flushed through the airtight chamber for 20 minutes at a flow rate of 3 LPM. The chamber oxygen level is monitored with an oxygen analyzer (MAXO_2_) (OM-25AE: Maxtec, Inc., Salt Lake City, UT). After the chamber is purged, the gas source is disconnected; the chamber is sealed and placed into a continuous flow CO_2_ incubator at 37°C. The gas in the chamber and the medium in the plates are changed every 24 hours by following the same procedure.

### 2.6. Positive Pressure

Positive pressure support is also a commonly employed strategy to support respiration in the face of respiratory failure. Positive pressure may be fixed (continuous positive pressure) or cycled (intermittent mandatory ventilation) depending upon the clinical situation and requirements of the patient. As with oxygen therapy, positive pressure ventilation can be damaging to the lung and airways, and strategies to minimize airway injury can be developed through *in vitro* modeling. Injury due to positive pressure can result from pressure, stretch, or both [25, 26], but, in the current experiments, the stress of pressure was evaluated independent of stretch.

To model the airway injury due to the effects of continuous positive airway pressure (CPAP), monolayers are exposed to the experimental conditions designed to model continuous positive pressure therapy using the incubator chamber. The desired gas concentrations are achieved by following the protocol as described above. The outlet of the chamber was attached to a Rüsch pressure manometer (Teleflexmedical, Inc., Duluth, GA) to achieve normoxia (21%  FiO_2_) and hyperoxia (60%  FiO_2_) with and without +20 cmH_2_O pressure for 24 or 72 hours. When the manometer reading reached the desired level and the temperature reading was 37°C, the inlet and outlet of the chamber were clamped and disconnected from the gas. The gas in the chamber and the medium in the plates were changed every 24 hours and the conditions again verified.

### 2.7. Biological Agents

Calu-3 monolayers grown at an air-liquid interface can be treated with biologically active and clinically relevant agents to assess the effects of treatment on cell function and the responses to oxygen and positive pressure. By varying doses and concentrations, dose-response relationships can be assessed for specific outcomes. Also, it is possible to compare the effects of apical (airway side) or basolateral (blood side) treatment of monolayers and explore potential differences between these modes of drug delivery. 

As is discussed below and in the results reported later, our laboratory has evaluated a number of clinically relevant compounds and medications to assess the effect of treatment on Calu-3 cell responses to various stresses. The agent of interest is layered onto the apical surface of the monolayer.

### 2.8. Perfluorochemicals

Mature Calu-3 monolayers treated with perfluorochemical liquid perflubron (PFB) were exposed to normoxic (FiO_2_ = 21%, FiCO_2_ = 5%, balance nitrogen) or hyperoxic (FiO_2_ = 95%, FiCO_2_ = 5%, balance nitrogen) conditions.

### 2.9. Surfactants

Calu-3 cell monolayers cultured at an air-liquid interface were treated apically with 140 *μ*L of normal saline, Lucinactant (Surfaxin; Discovery Laboratories, Inc.), or Beractant (Survanta; Abbott Laboratories, Inc.). Treated monolayers were exposed to normoxic (FiO_2_ = 21%, FiCO_2_ = 5%, balance nitrogen) or hyperoxic (FiO_2_ = 60%, FiCO_2_ = 5%, balance nitrogen) conditions using a modular environmental chamber for 24 or 72 hours in an incubator at 37°C.

### 2.10. Statistical Analysis

The experimental design used was an independent group method in which each experiment was performed on a unique monolayer. At least 6 samples were used for each group. Statistical analysis was performed using Statistical Package for the Social Sciences (SPSS, version 17) software. All data are expressed as mean ± SEM. Differences between conditions for each parameter were analyzed using two-way analyses of variance (ANOVA). Where applicable, one-way ANOVA were run across groups at each time interval (i.e., 24 and 72 hrs), and post-hoc analyses were done using Bonferroni comparisons. Significance was accepted with *P* < .05; *n* = at least 6 monolayers for each condition.

## 3. Results

### 3.1. Experiments with Various Concentrations of Oxygen

Calu-3 cells exposed to graded oxygen concentrations (21%, 40%, 60%, and 80%  FiO_2_) demonstrate cell dysfunction and inflammation in a dose-dependent manner [5]. TER decreased in a dose- and time-dependent manner, whereas cell viability was reduced only at 72 hours and in the 40%, 60%, and 80% FiO_2_ groups. IL-6 secretion was elevated in all hyperoxic groups at 24 hours, and both IL-6 and IL-8 levels were greater in the 40% FiO_2_ group compared with all other groups at 72 hours [5]. In this model, airway epithelial cells demonstrated profound concentration and time-dependent responses to hyperoxic exposure with respect to cell physiology, viability, histology, and secretion of inflammatory mediators.

### 3.2. Experiments with the Administration of Pressure

**Figure 3:**
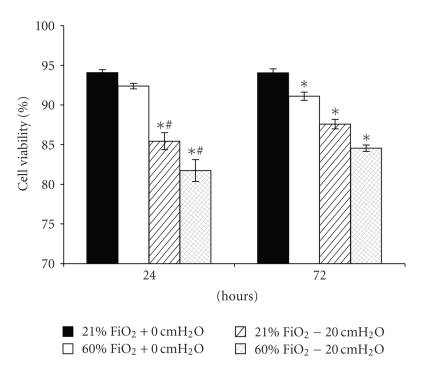
Cell viability for Calu-3 monolayers exposed to normoxia or hyperoxia with or without +20 cmH_2_O pressure. Cell viability decreased in both pressure-exposed groups (*P* < .001) but not in the control and hyperoxia only groups, which were not different from each other at 24 hrs (*P* > .05). By 72 hrs, cell viability was different in all groups, in which hyperoxia-exposed group was less than control group (*P* < .05) and greater than both pressure-exposed groups (*P* < .001). Data are mean ± SEM; *different from all other groups at the same time point; ^#^less than control and hyperoxia groups (*n* = 6 for each condition).

**Figure 4:**
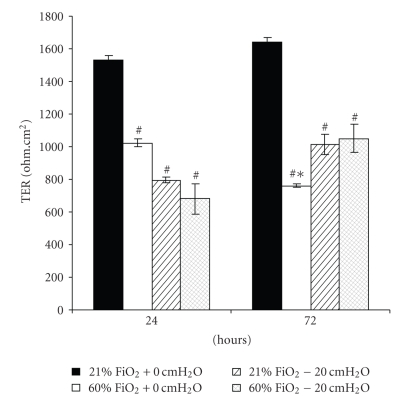
Transepithelial resistance (TER) was less than control in all treated groups (*P* < .001), independent of time. At 72 hrs, TER for the hyperoxia-exposed monolayers was less than that for the other treated groups (*P* < .001). Data are mean ± SEM; *different from all other groups at the same time point; ^#^ less than control at the same time point (*n* = 6 for each condition).

**Figure 5:**
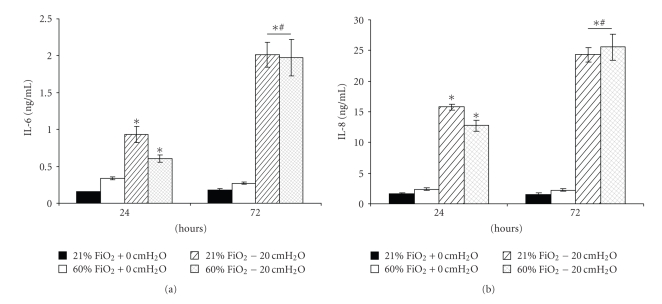
proinflammatory cytokines in ASF from normoxia- or hyperoxia-exposed Calu-3 monolayers with or without +20 cmH_2_O. Both IL-6 (a) and IL-8 (b) increased in pressure-exposed groups over time (*P* < .001) but not in control and hyperoxia alone groups. Pressure-exposed groups are greater than control and hyperoxia (*P* < .001) but not different from each other. Data are mean ± SEM; *greater than control and hyperoxia; ^#^greater than at 24 hrs. *n* = 6 for each condition.

**Figure 6:**
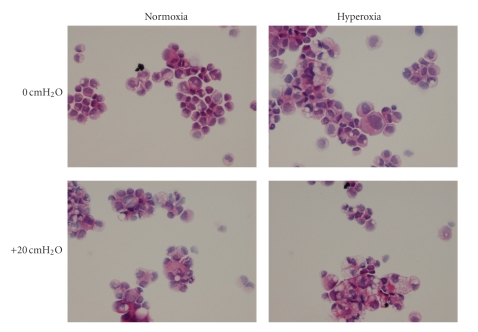
Representative histology. Cytospin preparations of Calu-3 cells after exposure to 72 hrs of normoxia or hyperoxia with or without +20 cmH_2_O pressure showed more swollen nuclei and intracellular vacuoles for pressure-treated cells than for the control and hyperoxia alone groups. Normoxia without pressure-exposed cells exhibited normal cellular histology.

Exposure to 21% FiO2 resulted in no changes in Calu-3 cell structure or function. In the presence of +20 cmH_2_O pressure for 24 or 72 hours, cell viability decreased in both pressure-exposed groups (*P* < .001) but not in the control and hyperoxia only groups, which were not different from each other at 24 hours (*P* > .05). By 72 hours, cell viability was different in all groups, in which the hyperoxia-exposed group was less than the control group (*P* < .05) and greater than both pressure-exposed groups (*P* < .001) (Figure 3). In Figure 4, transepithelial resistance (TER) was less than in the control group in all treated groups (*P* < .001). At 72 hours, TER for the hyperoxia-exposed monolayers was less than that for the other treated groups (*P* < .001). Similarly, both IL-6 (Figure 5(a)) and IL-8 (Figure 5(b)) increased in the pressure-exposed groups over time (*P* < .001) but not in the control and hyperoxia alone groups. The pressure-exposed groups were greater than the control and hyperoxia groups (*P* < .001) but not different from each other (*P* > .05). Cytospin preparations of Calu-3 cells showed normal cellular histology in the 21% FiO_2_ group at both time points, whereas the pressure- and hyperoxia-exposed groups showed more swollen nuclei and intracellular vacuoles (Figure 6).

### 3.3. Experiments with Pharmacological Agents

Calu-3 monolayers treated with either normal saline, Lucinactant, or Beractant were exposed to 60% FiO_2_ for 24 or 72 hours [24]. TER and cell viability were greater in both surfactant groups compared with saline; by 72 hours, Lucinactant cells had greater TER than Beractant cells. Both surfactant-treated groups secreted less IL-8 than saline, whereas Lucinactant cells secreted less IL-6 than both saline and Beractant cells. Histology indicated less injury with Lucinactant relative to Beractant and saline. Over 72 hours, Lucinactant was protective of Calu-3 cells compared with a Beractant and normal saline control [24].

Calu-3 cells treated with perflubron (PFB) and hyperoxia showed an increase in TER value, improved histology, decreased total protein secretion in ASF washings, and unaltered IL-8 secretion [13]. Cytomorphologic observations of PFB-treated Calu-3 cells indicated the presence of varying numbers of differently sized intracellular vacuoles during both normoxic and hyperoxic conditions. Calu-3 monolayers exposed to hyperoxia exhibited a loss of cellular integrity and decreased protein concentration and IL-8 level in ASF washings. In this study, PFB protects against hyperoxia-induced airway epithelial cell injury by promoting cellular integrity as well as cytologic modifications [13].

## 4. Discussion

In this paper, we present our experience using Calu-3 cells as a model for assessing the effects of oxygen concentration, positive airway pressure, and several clinically relevant pharmacological agents on various parameters of lung and airway injury. Under atmospheric conditions, Calu-3 cells have been widely used to model lung pathophysiology and therapeutics. Although immortalized, Calu-3 cells are of human derivation [8] and exhibit many characteristics of primary airway cultures. Calu-3 cells have been used as a model to examine transport [6, 27, 28] and metabolism of many therapeutic compounds [29] and particle-cell interaction studies [30–32]. Finally, studies regarding the absorption, metabolism, and distribution of inhaled corticosteroids have been performed with Calu-3 cell monolayers.

In the model of continuous positive airway pressure, in which Calu-3 monolayers were exposed to normoxia (control), hyperoxia, pressure (+20 cmH_2_O), or a combination of hyperoxia and positive pressure, a group effect for cell viability revealed that hyperoxia was less detrimental than pressure, but both insults in combination are more detrimental than either independently. At 24 hours, TER decreased acutely in all treated Calu-3 monolayers, and there appeared to be at least partial recovery in some groups by 72 hours. In the current experiments, the proinflammatory mediators IL-6 and IL-8 secretion increased over time for up to 72 hours for pressure groups. We can speculate that positive pressure triggered airway epithelial cells to secrete more proinflammatory mediators than hyperoxia alone, and the combination of positive pressure and hyperoxia did not appear to be additive. The lack of IL-6 and IL-8 secretion in response to hyperoxia was somewhat surprising but suggests that a higher FiO_2_ may be required to elicit a response in this model. Cell morphology of Calu-3 cells after exposure to 72 hours of normoxia or hyperoxia with or without +20 cmH_2_O pressure showed more swollen nuclei and intracellular vacuoles for pressure-treated cells than that for the control and hyperoxia alone groups, whereas normoxia-exposed cells exhibited normal cellular histology. These data suggest that Calu-3 monolayers are sensitive to hyperoxia and positive pressure, but that pressure may exert a greater injurious effect and cytokine response. These effects vary with the severity and duration of the stress.

 The current studies provide an important complement to this body of work by expanding the use of Calu-3 cells to include respiratory interventions commonly employed in critical care medicine. The experiments show that Calu-3 cells can be exposed and respond to oxygen, static, and cyclically delivered positive pressure in clinically relevant ways. The responses of Calu-3 monolayers, particularly in terms of the IL-6 and IL-8 responses, reflect those responses seen *in vivo*. In addition, these responses can be modulated with clinically relevant treatments. In our studies, both Lucinactant and perflubron attenuated Calu-3 cell responses to hyperoxia. These findings are notable given the importance of surfactant replacement therapy in the newborn intensive care unit and the role of proinflammatory cytokines such as IL-6 and IL-8 in the pathogenesis of chronic respiratory disorders such as bronchopulmonary dysplasia.

In summary, Calu-3 air-liquid interface cell culture in an environmental chamber is a sensitive and efficient model to simulate clinical situations and for screening respiratory drug applications. This cell model is useful for the study of human respiratory diseases, airway injury related to oxygen toxicity, and positive mechanical ventilation, and the evaluation of novel therapies. In many cases, isolated cell preparations allow for very careful control of cell environment (i.e., the air-liquid interface culture enables the direct sampling and measurement of luminal airway secretions in the absence of systemic factors). However, it should be noted that the absence of systemic inflammation, circulatory factors, and other paracrine systemic influences is a potential limitation of the isolated cell system and should be carefully considered for certain investigations.
